# Efficacy of far infrared functional glasses in the treatment of meibomian gland dysfunction‐related dry eye

**DOI:** 10.1002/mco2.507

**Published:** 2024-03-22

**Authors:** Lei Tian, Yihan Guo, Silu Wang, Zhongying Li, Ningli Wang, Ying Jie

**Affiliations:** ^1^ Beijing Institute of Ophthalmology Beijing Tongren Eye Center Beijing Tongren Hospital Capital Medical University, Beijing Ophthalmology & Visual Sciences Key Laboratory Beijing China; ^2^ Beijing Advanced Innovation Center for Big Data‐Based Precision Medicine Beihang University and Capital Medical University Beijing China

**Keywords:** dry eye, far infrared, functional glasses, meibomian gland dysfunction

## Abstract

Meibomian gland dysfunction (MGD)‐related dry eye disease (DED) is a significant subtype of DED. In this research, we investigate the effectiveness of far infrared (FIR) functional glasses in the treatment of MGD‐related DED. According to the TFO DEWS II diagnostic criteria, 61 eyes with MGD‐related DED were included. All participants wore functional FIR glasses throughout the day for a period of 4 weeks and were followed up three times during the treatment. All subjects were followed up thoroughly in accordance with the DED clinical examination procedure. Ultimately, the treatment's impact was assessed. We found the Visual Analogue Scale and Ocular Surface Disease Index scores after FIR treatment were significantly lower than the baseline values (*p* < 0.05). Compared with the baseline, fluorescein tear breakup time and corneal fluorescein staining score after FIR treatment were significantly improved (*p* < 0.05). The eyelid margin signs, meibum quality, and meibomian gland expressibility after the 4‐week treatment were significantly better than those at baseline (*p* < 0.05). We can see that wearing the FIR functional glasses significantly relieves the symptoms and signs of patients. We believe FIR therapy could be considered as a new method of MGD‐related DED.

## Graphical abstract

In this clinical trial, 61 patients with MGD‐related DED wore FIR‐functional eyeglasses throughout the day and underwent a comprehensive eye assessment at the trial's end. Significant improvements were observed in the symptoms, clinical signs, and meibomian gland function of patients with MGD‐related DED. These findings suggest that FIR therapy could emerge as a novel approach for MGD‐related DED.

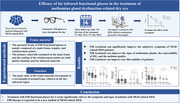

## 1 INTRODUCTION

Dry eye disease (DED) is a multifactorial disease of the tears and ocular surface. Accompanied by increased osmolarity of the tear film and inflammation of the ocular surface, leading to discomfort, visual disturbance, and tear film instability, and may result in potential damage to the ocular surface. In recent years, DED has become one of the most common ocular surface diseases affecting people's quality of life. The prevalence of DED varies from 5.5 to 33.7% worldwide, and the prevalence in China is about 21–30%.^1^, ^2^, ^3^ Consequently, the proactive exploration of the etiological factors behind the dry eye and the quest for more convenient and effective treatment methods have assumed paramount significance.

Evaporative dry eye is the most common subtype of DED, which is mainly caused by meibomian gland dysfunction (MGD).^4^, ^5^ According to the International Workshop on Meibomian Gland Dysfunction (IWMGD), MGD is a chronic, diffuse abnormality of the meibomian glands, commonly characterized by terminal duct obstruction and/or qualitative or quantitative changes in the glandular secretion, which can lead to changes in the tear film, eye irritation symptoms, clinically evident inflammation, and ocular surface diseases.^6^ MGD is a complex condition with multiple underlying mechanisms, including eyelid inflammation, conjunctival inflammation, corneal damage, microbial changes, and tear film instability.^7^, ^8^ Any combination of these factors can lead to MGD. It has been indicated that MGD and DED are interconnected, forming a double vicious circle,^7^ in which eyelid gland blockage, dropout, and inflammation are the core mechanisms leading to MGD‐related DED. Clinical treatments of MGD‐related DED are mainly based on topical medication and physiotherapy, such as hot compresses and massage, which can alleviate the associated clinical symptoms to some extent, but they are often costly, cumbersome, and mostly fail to achieve the desired therapeutic effect.^9^ Recently, researchers have been working on new options for the treatment of MGD‐related DED.

Far infrared (FIR) is an infrared ray with the longest wavelength and possesses key physical properties such as emissivity, permeability, absorption, and resonance.^10^ Generally, FIR is defined as infrared rays with a wavelength of 2.5 µm or more. FIR with a vibrational frequency similar to human cell molecules can produce a significant thermal effect, increasing the temperature of the inner layer of skin, widening capillaries, and improving blood microcirculation,^11^ thus increasing metabolism.^12^ Furthermore, numerous studies have indicated that FIR radiation can impact various physiological processes in the human body through nonthermal effects. These effects involve enhancing the production of nitric oxide (NO), elevating calcium‐regulated protein (CaM) levels, and reducing the production of cyclooxygenase‐2 and prostaglandin E2, thus affecting processes related to anti‐inflammation, pain relief, metabolic regulation, and apoptosis.^13^ At present, many products based on the FIR principle have been widely used in the medical field, such as gloves to treat Raynaud's syndrome^14^ and blankets to improve sleep.^15^ With the development of technology, the use of FIR for the treatment of visual fatigue^12^ and pseudomyopia^16^ has attracted a lot of interest in the healthcare field. Therefore, we hypothesize that FIR radiation, leveraging its unique physical properties, can exert both thermal and nonthermal effects, working in synergy within the double vicious circle of MGD and dry eye. This combination of effects could offer distinct advantages in addressing these conditions.

In this trial, we designed a glass frame, which can continuously emit FIR at a wavelength of about 8 um. We applied these eyeglasses for the treatment of MGD‐related DED and conducted research to evaluate its effectiveness, safety, and underlying therapeutic mechanisms. Our goal is to offer a comfortable, convenient, affordable, effective, and safe treatment option for dry eye patients that can be easily incorporated into their daily routines by wearing these glasses.

## 2 RESULT

### 2.1 Characteristics of the participants

**FIGURE 1:**
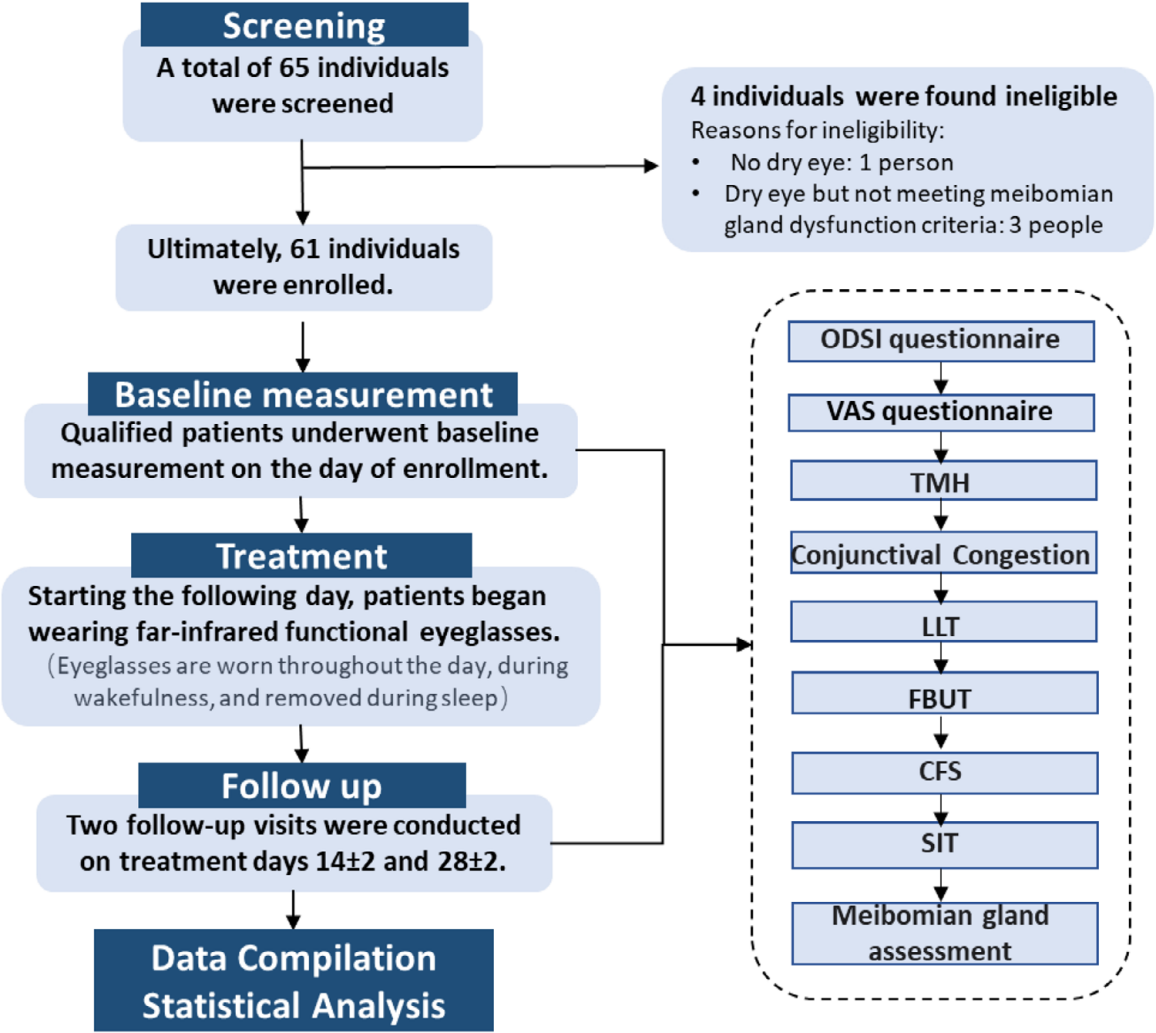
Flow chart of the cohort establishment.

After careful screening, the study involved a total of 61 eyes from 61 patients diagnosed with MGD‐related DED, comprising 50 females and 11 males. The mean age of the patients was 58.67 ± 8.83 years, and the age range was 34−74 years. According to the Expert Consensus on Diagnosis and Treatment of Meibomian Gland Dysfunction in China (2017),^17^ patients' severity of MGD was graded. Among the participants, 12 individuals (19.67%) were categorized as mild, 43 (70.49%) as moderate, and six (9.84%) as severe (Table 1). All patients completed follow‐up and underwent an entire course of FIR treatment and eye examination (Figure 1).

**TABLE 1:** Patient characteristics of the cohort.

	Total (M ± SD or *N*)
Patients (eyes)	61
Age	58.67 ± 8.83
Grade of MGD	
Mild	12
Moderate	43
Serious	6

Abbreviation: MGD, meibomian gland dysfunction.

### 2.2 Subjective symptoms

The scores of the Ocular Surface Disease Index (OSDI) questionnaire and Visual Analogue Scale (VAS) questionnaire can represent patients' chief sensations of dry eye symptoms. After 4 weeks of FIR treatment, both OSDI and VAS indicators had sustained significant improvement, with significant differences from baseline values (*p* < 0.01), and with the exception of the OSDI environmental trigger (*p* = 0.116), the difference was also statistically significant (*p* < 0.05) when comparing scores at 2 and 4 weeks of treatment (Table 2). These results indicate that FIR treatment can significantly improve the subjective dry eye symptoms in patients.

**TABLE 2:** Subjective symptoms during FIR treatment at three follow‐up visits.

	V0 (M ± SD)	V1 (M ± SD)	V2 (M ± SD)	*P_1_*	*P_2_*	*P_3_*
OSDI score						
Total score	36.90 ± 11.79	29.66 ± 11.85	24.93 ± 11.81	<0.001	<0.001	0.001
Vision‐related function	37.52 ± 14.78	32.38 ± 13.06	27.58 ± 12.84	0.005	<0.001	0.005
Ocular symptoms	34.94 ± 16.10	28.62 ± 18.11	22.47 ± 14.01	0.019	<0.001	0.006
Environmental triggers	36.20 ± 16.59	25.41 ± 16.82	22.68 ± 14.12	<0.001	<0.001	0.116
VAS						
Burning/stinging	19.82 ± 23.39	14.89 ± 18.89	12.49 ± 17.15	0.010	0.002	0.005
Itching	34.31 ± 29	26.75 ± 26.21	23.20 ± 23.82	<0.001	<0.001	0.003
Foreign body sensation	35.44 ± 28.99	28.8 ± 26.23	23.61 ± 22.31	<0.001	<0.001	<0.001
Blurring of vision	57.9 ± 22.54	45.69 ± 22.05	35.64 ± 19.59	<0.001	<0.001	<0.001
Eye discomfort	57.3 ± 25.36	46.41 ± 23.4	37.79 ± 20.90	<0.001	<0.001	<0.001
Photophobia	41.61 ± 33.08	32.87 ± 29.13	29.67 ± 25.22	<0.001	<0.001	<0.001
Pain	19.02 ± 24.9	14.89 ± 18.89	12.39 ± 19.06	0.032	0.001	0.014

Abbreviations: OSDI, Ocular Surface Disease Index; VAS, Visual Analogue Scale.

Statistical significance is set at *p*<0.05 and expressed as *P_1_*: 2 weeks versus baseline, *P_2_*: 4 weeks versus baseline, *P_3_*: 4 weeks versus 2 weeks.

### 2.3 Indicators of dry eye signs

Clinical indicators of DED can be closely correlated with the ocular condition of dry eye patients. Dry eye indicators and their clinical significance are related as follows: (1) tear film stability is assessed using fluorescein tear film breakup time (FBUT); (2) tear secretion capacity is assessed using tear meniscus height (TMH) and Schirmer's tear test (SIT); (3) evaluation of tear film lipid layer is performed through lipid layer thickness (LLT).

The patient's FUBT after 4 weeks of treatment (3.68 ± 0.66) was statistically significantly higher than that of the baseline values (3.32 ± 0.76) (*p* < 0.001). There was no significant difference in the patients' TMH and SIT before and after the FIR treatment (*p* = 0.810, *p* = 0.892). There was an increasing tendency of LLT in the three follow‐up visits, but the difference was not statistically significant (*p* = 0.216, *p* = 0.401, *p* = 0.816). Compared with baseline values, there is a statistically significant difference in the conjunctival congestion degree and corneal fluorescein staining (CFS) score after 4 weeks of FIR treatment (*p* = 0.012, *p* < 0.001) (Table 3). It is evident that employing FIR glasses has significantly improved the stability of the patient's ocular surface and increased the thickness of the tear film lipid layer.

**TABLE 3:** Results of dry eye indicators at three follow‐up visits.^9^

	V0 (M ± SD)	V1 (M ± SD)	V2 (M ± SD)	*P_1_*	*P_2_*	*P_3_*
FBUT	3.32 ± 0.76	3.57 ± 0.77	3.68 ± 0.66	0.001	<0.001	0.063
TMH	0.19 ± 0.08	0.19 ± 0.08	0.19 ± 0.08	0.392	0.552	0.810
SIT	7.40 ± 5.84	7.35 ± 4.82	7.22 ± 5.03	0.103	0.263	0.892
LLT	63.89 ± 23.72	67.67 ± 22.43	67.9 ± 23.84	0.216	0.401	0.816
CFS	1.75 ± 1.36	1.38 ± 1.32	1.10 ± 1.33	0.001	<0.001	0.034
Conjunctival congestion	2.35 ± 0.54	2.34 ± 0.55	2.16 ± 0.41	0.012	0.012	0.616

Abbreviations: CFS, corneal fluorescein staining; FBUT, fluorescein tear breakup time; LLT, lipid layer thickness; SIT, Schirmer I test; TMH, tear meniscus height.

Statistical significance is set at *p*<0.05 and expressed as *P_1_*: 2 weeks versus baseline, *P_2_*: 4 weeks versus baseline, *P_3_*: 4 weeks versus 2 weeks.

### 2.4 Meibomian gland assessment

**FIGURE 2:**
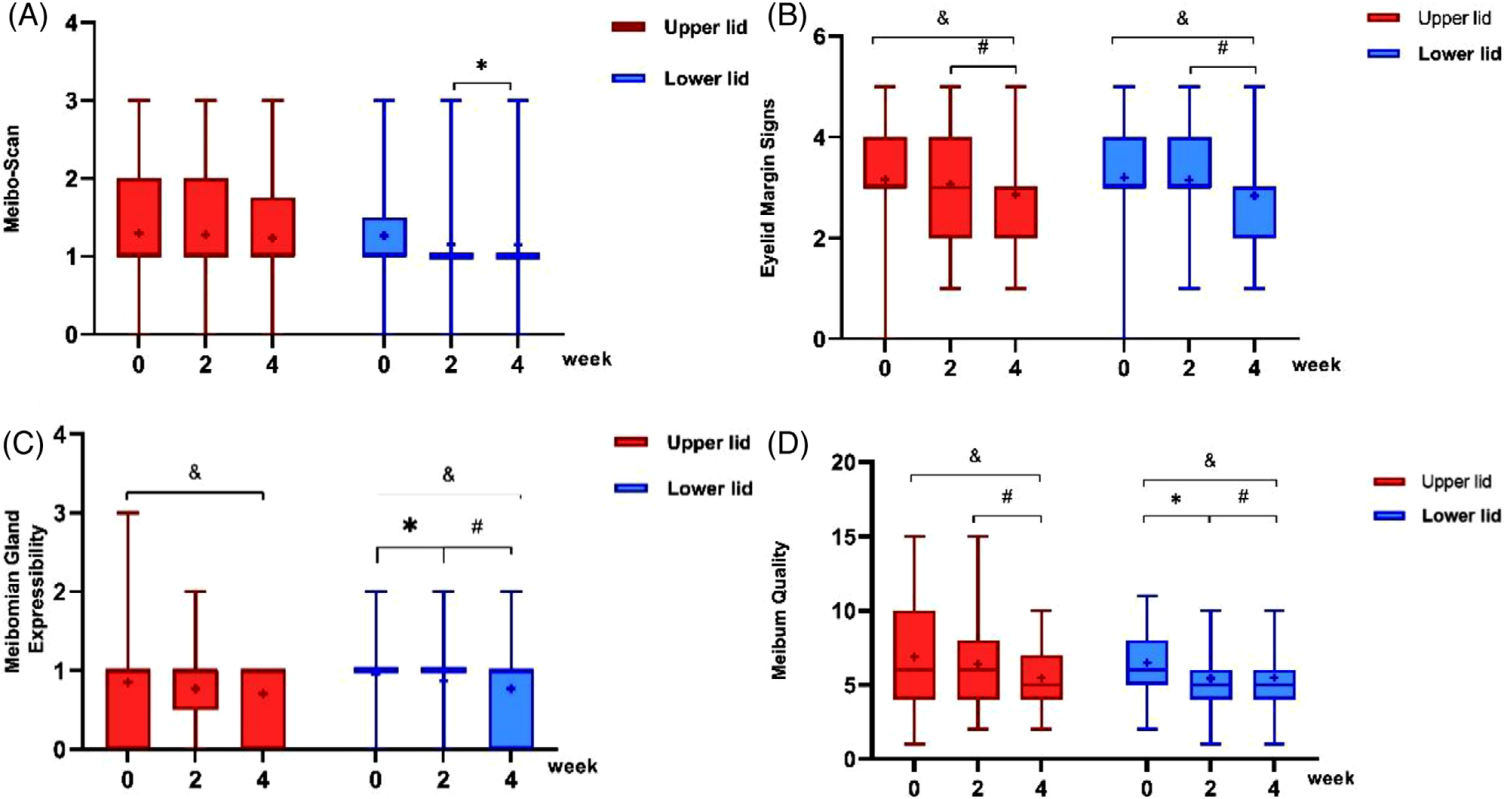
Meibomian gland evaluation results at three follow‐up visits. (A) Meibo‐Scan scores of the upper and lower eyelids, (B) the scores of eyelid margin signs of the upper and lower eyelids, (C) the scores of meibomian gland expressibility of the upper and lower eyelids, and (D) meibum quality scores of the upper and lower eyelids.

A comprehensive assessment of patients' meibomian gland parameters is beneficial for determining the functionality of their meibomian glands. As demonstrated in Figure 2, the patient's eyelid margin signs and meibum quality in both upper and lower lids after 4 weeks of treatment were significantly better than the baseline values (*p* = 0.009, *p* < 0.001; *p* = 0.001, *p* < 0.001) and the scores at 2 weeks (*p* = 0.017, *p* < 0.001; *p* < 0.001, *p* < 0.001). After a 4‐week treatment period, there was a notable decrease in the scores for meibomian gland expressibility (both upper and lower lids) compared with baseline values (*p* = 0.049, *p* = 0.001) (Figure 2). The use of FIR glasses has resulted in substantial improvements in the patients' meibomian gland signs and meibum secretion, effectively alleviating their MGD condition.

### 2.5 Safety examinations

The analysis of the elemental composition of the FIR material within the eyeglass frame, conducted through a scanning electron microscope (SEM), revealed that no harmful elements or heavy metals such as As and Hg were detected. The main crystalline phases identified in the sample, determined by X‐ray diffraction (XRD), consisted of compounds commonly found in nature, displaying no significant toxicity. The spectral radiance of the sample adhered to the black‐body radiation law across the entire wavelength range, without any indication of abnormal radiation peaks, signifying the absence of potentially harmful irregular light radiation.

Moreover, the materials contained within the FIR eyeglass frame underwent comprehensive analysis by the Sichuan Institute for Food and Drug Control. This analysis confirmed that the materials do not irritate the skin, do not induce sensitization, and do not exhibit cytotoxic effects on cells (Report numbers: QX2021P04989‐10b‐01, QX2021P04989‐10a‐01, QX2021P04989‐05a‐01).

Throughout the 4‐week period of FIR treatment, no abnormal alterations were observed in patients' visual acuity (VA), intraocular pressure (IOP), or the condition of the corneal surface (Table 4). Slit lamp examinations revealed that there were no indications of corneal opacity or corneal edema, results for both keratic precipitates and Tyndall syndrome were negative, and no significant changes were observed in the lens or vitreous. Two experienced ophthalmologists meticulously analyzed fundus photographs of the patients. None of the patients displayed pathological changes in the fundus, and key fundus structures, including the optic cup, optic disc, central retinal arteries and veins, macula, optic nerve head, and cup‐disc ratio, remained unchanged.

**TABLE 4:** Visual acuity, intraocular pressure, and corneal surface conditions during follow‐up.

	VO	V1	V2	*P_1_*	*P_2_*	*P_3_*
VA	0.66 ± 0.32	0.71 ± 0.34	0.72 ± 0.32	0.097	0.024	0.777
IOP	15.34 ± 2.61	15.35 ± 2.93	14.94 ± 2.96	0.900	0.112	0.069
CFS	1.75 ± 1.36	1.38 ± 1.32	1.10 ± 1.33	0.001	<0.001	0.034

VA, visual acuity; IOP, intraocular pressure; CFS, corneal fluorescein staining.

Statistical significance is set at *p*<0.05 and expressed as *P_1_*: 2 weeks versus baseline, *P_2_*: 4 weeks versus baseline, *P_3_*: 4 weeks versus 2 weeks.

## 3 DISCUSSION

DED is currently one of the most common ocular diseases, with nearly 300 million dry eye patients in China.^18^ As the pathogenesis of dry eye continues to be explored, it has been realized that MGD is the most common cause of dry eye onset. As reported, the prevalence of MGD ranges from 46.2 to 69.3% in Asians among the elderly.^19^ How to standardize and effectively treat MGD‐related DED has been a hot topic in the field of ophthalmology.

When age, hormone levels, and medications cause hyperkeratosis of the meibomian duct epithelium and increased lid ester viscosity, the terminal ducts of the meibomian glands become obstructed, leading to glandular abscission, atrophy, and decreased secretion.^20^ In this case, the quality or quantity of the lipid delivered to the tear film is decreased, which causes a vicious cycle of tear film instability, excessive evaporation, and ocular surface inflammation,^20^ resulting in a series of dry eye symptoms.^20^ Therefore, improving the flow of lid secretions, opening the terminal ducts of the lid gland, and reestablishing lacrimal film stability in patients with MGD are the major priorities in the treatment of MGD.^21^

Current treatments for MGD mainly include antibiotics, nonsteroidal anti‐inflammatory drugs, hormonal therapy, control of crepuscular mite infections, hot compresses, and intense pulsed light (IPL) therapy.^22^, ^23^ In recent years, it turns out that more and more research has focused on the use of physiotherapy to treat MGD, with some success. With advances in technology, the therapeutic effects of FIR have attracted much attention. Clothing and blankets made from FIR materials have been shown to improve sleep quality,^15^ treat arthritis in the hands,^14^ cause body fat reduction, and improve skin elasticity.^24^ It has been reported that FIR treatment can inhibit the growth of tumors in mice, treat diseases related to blood vessels,^15^, ^25^ control pseudomyopia, and improve visual fatigue.^12^, ^16^ As far as we know, there were no studies that applied FIR to dry eye treatment. Therefore, we innovatively propose a new treatment for MGD‐related DED and investigate its effectiveness.

In our study, the chief observations were as follows: first, FIR treatment can significantly improve the subjective symptoms of MGD‐related DED patients. Second, FIR treatment can improve the signs of meibomian glands, the expressibility of MG, and the meibum quality. Third, FIR treatment can improve the tear film stability of patients. What is more, after FIR treatment, there were no abnormal changes in patients’ IOP, uncorrected VA, and fundus examination, indicating that FIR treatment is safe and will not cause adverse effects on patients’ eyes.

How FIR has a therapeutic effect on the human body has been a hot topic of research. On the one hand, FIR vibrates at frequencies close to those of human cell molecules, can penetrate up to 1.5 inches (nearly 4 cm) below the skin, producing a significant thermal effect that increases the temperature of the inner layers of the skin, enlarges capillaries and improves blood microcirculation,^11^ thereby enhancing metabolism.^10^ On the other hand, some scientists have found that nonthermal FIR therapy increases skin blood flow in rats^11^ and that repeated FIR treatment upregulated endothelial NO synthase expression.^26^ In addition, Yu et al.^11^ suggested that the mechanism by which FIR therapy promotes cutaneous blood flow is closely related to the l‐arginine pathway. Therefore, the nonthermal effects of FIR can also have biological effects on humans.

The FIR functional glasses developed in this study are equipped with a reinforcing point at the frame, which is mainly composed of rutile, tourmaline, and other materials. It has been documented that titanium dioxide (TiO_2_) has good FIR properties^27^ and has the effect of improving blood circulation and stimulating the lymphatic system.^27^ Based on the analysis of the infrared spectra of TiO_2_ materials, it is clear that the peak at 7.3 µm is attributed to the titanium‐hydrogen (Ti‐H) bond^28^ and the absorption peak at about 11.8 µm reveals the titanium‐oxygen (Ti‐O) bond.^29^ Tourmaline can emit FIR electromagnetic waves with a wavelength of 4–14 µm, thus accelerating human blood circulation, promoting metabolism, and improving human microcirculation.^30^ With good FIR properties, maifan stone can emit FIR waves of 8–15 µm^31^ and is now suggested to be used in the medical field.^32^

In the current study, we found that wearing FIR glasses during the treatment (within 4 weeks) was effective in increasing the meibomian gland expressibility and improving the meibum quality in patients with MGD‐related DED. There was also a significant improvement in eyelid margin signs. We believe that this significant improvement in meibomian gland function and meibum quality is closely related to the thermal effect of FIR. Liu et al.^33^ and Bäumler et al.^34^ demonstrated that the thermal effect has a significant effect on the treatment of MGD, which is consistent with our findings. Normally, when the ocular surface and eyelid temperature vary between 33 and 37°C, the meibum can remain in a fluid state.^35^ The temperature at which the meibum changes from a semi‐solid to a liquid state is known as the phase change temperature. It has been shown that the composition of meibum in MGD patients changes, with increased concentrations of sphingoid metabolites, acrylamide, and decreased concentrations of lipids, esters, and free sterols,^36^ led to increased viscosity and phase change temperature.^37^, ^38^ In addition, the potential involvement of microbes (*Staphylococcus* spp, *Bacillus oleronius*, *Propionibacterium acnes*, and the *Demodex* species described above) contributes to the pathology of MGD‐related DED by increasing the melting temperature of meibum.^39^ These changes ultimately lead to meibomian gland terminal duct obstruction and reduced lipid delivery to the tear film. These FIR glasses emit regular and sustained FIR light around the patient's eyelids, with enough FIR energy that can exert a vibrational and rotational pattern of motion in the bonds that form molecules (including water molecules), resonating with cellular frequencies.^10^ In this way, FIR can transmit energy to subcutaneous tissue 2–3 cm deep, producing a significant thermal effect that raises the temperature of the inner layers of the skin.^15^, ^40^ We know that the temperature of the eyelid area affects the physical properties of the meibomian gland: as the temperature increases, the meibum becomes easier to flow and the viscosity of which decreases,^41^, ^42^ hereby ameliorates meibomian gland obstruction and thus enhances its ability to secrete.

Our study showed that FIR treatment significantly improved the FUBT, suggesting that FIR treatment could significantly improve the stability of the tear film. Previous studies have shown that meibomian glands can transport meibum outwards, which could be coated on the ocular surface during a blink, forming the tear film's outer layer, which can block the contact between the water layer and the air, thereby reducing evaporation, which is essential to maintain the stability of the tear film. Due to the thermal effect of FIR, the quality or quantity of meibum delivered to the ocular surface is improved, so that the LLT is thickened and the stability is improved, which prevents the rapid evaporation of tears in the ocular surface and thus improves the stability of the tear film.^43^, ^44^

We observed that after 4 weeks of FIR functional glasses treatment, patients' OSDI and VAS scores were significantly reduced, and patients' subjective symptoms such as dry burning/stinging sensation, dry eyes, and itching were significantly improved. In a study by Hu and Li,^45^ patients' ocular itching symptoms improved significantly during FIR treatment for two to three. Yokoyama and Oku demonstrated that infrared radiation has a good analgesic effect, which is consistent with our findings.^46^ Studies have shown that FIR dilates capillaries and improves blood circulation,^15^ thus potentially accelerating the relief of ocular fatigue and also providing improvement in subjective symptoms in patients with dry eyes. Yu et al.^11^ suggested that FIR therapy promotes skin blood flow through a mechanism closely related to the l‐arginine pathway, thus the improvement in patients’ subjective symptoms may be associated with the nonthermal effects of FIR.

Vatansever et al.^10^ suggest that the nonthermal effect of FIR acts similarly to low‐level laser (light) therapy (LLLT), whereby mitochondrial chromophores such as cytochrome *C* oxidase (unit IV of the mitochondrial respiratory chain) absorb near infrared (NIR). This photon absorption may activate the enzyme by photodissociating the inhibitory molecule NO from the copper B site.^47^ As a result of the loss of NO, electron transfer, oxygen consumption, and adenosine triphosphate (ATP) production increase rapidly, and mitochondrial membrane potential also increases significantly, which causes a transient burst of reactive oxygen species (ROS).^48^ The signaling pathway is activated by ATP, NO, and ROS, leading to the activation of transcription factors (e.g. nuclear factor kappa‐B),^49^ resulting in long‐term effects on the tissue (healing, anti‐inflammation, and pain relief).^50^ However, this explanation is still hypothetical and we need further probing to test this hypothesis.

Furthermore, the inflammatory response is a common mechanism in the pathogenesis of various types of dry eye.^51^ Bron et al.^52^, ^53^ proposed the tear gradient theory, according to which tear evaporation leads to an increase in solute concentrations, particularly of proinflammatory proteins in the lid gland. Studies have shown that the photothermal effect of IPL can reduce inflammation by reducing the levels of pro‐inflammatory mediators reaching the lid gland through the induced ablation of small blood vessels around the eyelid margin,^54^ and we speculate that the thermal effect produced by FIR would act in a similar way on the inflammatory response in patients with dry eye, causing an improvement in a range of signs and symptoms. However, the exact mechanism of its action remains unclear and needs to be further explored.

Furthermore, we have consistently prioritized the safety of FIR glasses. Currently, multiple studies have confirmed the safety of FIR therapy in treating conditions such as extremities lymphedema,^55^ breast cancer‐related lymphedema,^56^ primary dysmenorrhea,^57^ and rotator cuff diseases.^58^ These studies have affirmed that FIR therapy while boosting blood circulation, enhancing cellular metabolism, and alleviating pain, poses no adverse effects on the human body. Throughout our research, we have conducted comprehensive safety assessments during follow‐up examinations. These assessments encompass VA tests, IOP measurements, fundus photography, and detailed examinations of various ocular structures, including the eyelid skin, conjunctiva, cornea, iris, lens, and vitreous, performed with a slit lamp. Notably, our study revealed no abnormal reductions in VA, and IOP consistently remained within the normal range without significant fluctuations. Moreover, CFS scores displayed a significant increase, signifying the FIR not only does not damage the cornea but may even lead to improvements. Patients did not report any adverse reactions such as redness, numbness, or discomfort in the eyelids and surrounding skin. Detailed slit lamp examinations detected no significant abnormalities in the anterior ocular segment, and no abnormal alterations were observed in the patient's fundus. These collective observations provide substantial evidence supporting the safety of FIR functional glasses as a treatment modality.

So far, there are very few studies on FIR rays for the treatment of dry eyes, therefore our study innovatively proposes a new method of dry eye treatment, confirms its safety, and tests its effectiveness. Of course, our study has some limitations; first, the sample size is small, and it still needs to be expanded to demonstrate the generalizability of the study. Second, our study was only 4 weeks, which is a little short. Third, in further studies, we should add a control group to enhance the validity of the results. And, further studies are still needed to demonstrate the specific mechanisms by which FIR works on MGD‐related DED.

## 4 CONCLUSION

FIR functional glasses can significantly improve the subjective symptoms and tear film stability, as well as meibomian gland signs, expressibility, and meibum quality of patients with MGD‐related DED. The use of FIR functional glasses is therefore expected to be a new treatment for MGD‐related DED.

## 5 MATERIALS AND METHODS

### 5.1 Study design

This study is interventional research (clinical trial registration number: ChiCTR2300079099), where participants were enrolled in a naturally occurring random sequence and served as their own controls in a before‐and‐after study. The main text was described according to the Transparent Reporting of Evaluations with Nonrandomized Design (TREND) statement available from: https://www.cdc.gov/trendstatement/index.html.

### 5.2 Participants

This study screened a total of 65 individuals diagnosed with MGD‐related DED at the Cornea Clinic of Beijing Tongren Hospital, Beijing, from October 2022 to December 2022. To prevent potential correlations between the two eyes of the same patient from interfering with the results, the right eye was uniformly used for analysis.

#### 5.2.1 Inclusion and exclusion criteria

Inclusion criteria were as follows: When patients meet the diagnostic criteria for both DED and MGD, they are identified as MGD‐related DED.

DED was diagnosed based on the TFO DEWS II diagnostic methodology report (2017), which implies: (1) symptoms: OSDI questionnaire ≥13 scores or dry eye questionnaire (DEQ‐5)≥6; (2) at least one positive result of the markers of homeostasis listed below: FBUT ≤10 s, Schirmer I test (without surface anesthesia) ≤10 mm/5 min, or CFS: >5 corneal spots.^59^

According to the International Workshop on MGD: Report of the Diagnosis Subcommittee(2011), MGD can be diagnosed as follows: (1) clinical signs: meibomian gland dropout, altered meibomian gland secretion, and changes in lid morphology; (2) uncomfortable global symptoms such as redness and swelling, itching, irritation, soreness.^60^

Key exclusion criteria were as follows: (1) participated in another clinical trial within the last 2 months; (2) severe systemic autoimmune diseases; (3) ocular diseases other than DED, including eye trauma, pterygium, active blepharitis, ocular/periocular malignancy; (4) active ocular allergies or existing drug and food allergies; (5) clinically relevant slit‐lamp findings or abnormal lid anatomy; (6) intraocular surgery or ocular laser surgery within 6 months; (7) pregnant, breastfeeding, or planning for pregnancy during the trial period.

#### 5.2.2 Sample size

In this study, the primary outcome measure is the VAS score for dryness. Drawing from preliminary research findings, the effect size is estimated at 1.6, with a corresponding standard deviation of 2.89. The research objective includes achieving a statistical power of over 80%, with a significance level set at 0.05. Consequently, a minimum enrollment of 36 participants is deemed necessary. Accounting for a potential loss to follow‐up rate not exceeding 20% during short‐term follow‐up, a total of at least 45 participants should be included to ensure robust statistical precision.

#### 5.2.3 Assignment method

A single‐group before‐and‐after test was done, so that there was no specific assignment method.

When evaluating the degree of MGD in the 61 eyes included in this study, our deliberate effort to select patients with mild‐to‐moderate dysfunction (including 12 (19.67%) mild and 43 (70.49%) moderate individuals). This intentional selection was made to enhance the homogeneity of our study sample and reduce potential confounding factors associated with varying degrees of dysfunction.

### 5.3 Interventions

#### 5.3.1 Setting

Subjects were given the functional FIR glasses to wear throughout the day (put on when awake, take off when asleep) for a period of 4 weeks, during which there were approximately three follow‐up visits: V0: first day of the treatment, V1: day 14 ± 2, V2: day 30 ± 2. At the end of the treatment, the effect of the treatment is evaluated.

#### 5.3.2 FIR functional glass treatment

FIR functional glass frame (YDkai Technology Co, Shenzhen, Beijing) is designed to enhance the self‐recovery and regulation of the eyes by means of wearing it, and to effectively prevent, relieve, and control eye fatigue, dry eyes, astringent, eyes, and other eye discomfort symptoms.

The spectacle frame of FIR Functional glasses is mainly composed of a main frame, temples, and reinforcement points (refer to Appendix 1; Figure S1). The primary materials contained in the main frame and the coating of the reinforcement points are both plastic titanium and FIR material. However, the content of the FIR material in the coating of the reinforcement points is higher than that of the main frame. The FIR material consists of six minerals in total. Using an X‐ray diffractometer (Ultima IV), Professor Gu Qiang and his team from the University of Science and Technology Beijing performed phase analysis on the samples. According to the analysis of XRD results, FIR materials consist of six minerals: (A) rutile (TiO_2_), (B) tourmaline (magnesium tourmaline (Na_0.8_Mg_3_Al_6_B_3_O_9_Si_6_O_18_(OH)_4_), iron tourmaline (NaFe_3_Al_6_ (BO_3_)Si_6_O_18_ (OH)_4_), calcium magnesium tourmaline (CaMg_3_ (Al, Mg)_6_ (BO_3_)Si_6_O_18_ (OH)_4_); (C) iron powder (Magnetite (Fe^2+^Fe^2+^O_4_), quartz, iron titanium oxide (Fe_2.5_Ti_0.5_)_1.04_O_4_), hematite (Fe_2_O_3_)); (D) bentonite ((Faujasite‐Na)(Na_2_Al_2_Si_4_O_12!_8H_2_O), (Faujasite‐K)(K_48.2_Al_48.2_Si_143.8_O_384!_3H_2_O)); (E) Kaolin (mullite (Al_6_Si_2_O_13_)); (F) mixed powder (barium‐iron oxide). Meanwhile, Multiple energy dispersive X‐ray spectroscopy measurements were made for each sample using a machine SEM. In summary, all elements contained in the samples are common elements in nature, and no harmful elements and heavy metal elements such as Arsenic and Mercury have been found.

The frame is continuously irradiated with FIR rays in the 5–18‐micron wavelength range, allowing the frame to interact with the body by emitting FIR with a wavelength of about 8 microns. As we all know, material emissivity is an essential parameter in describing the properties of physical thermal radiation. The normal total emissivity refers to the ratio of the normal direction radiation emission to the surface of a thermal emitter to the normal radiation emission of a blackbody at the same temperature within the wavelength range of 0–∞. Based on Stefan–Boltzmann law, the radiated power of some materials is $P\;=\;\varepsilon A\sigma T^{4}$, where *ε*, *A*, *σ* is the total normal emissivity of the sample, the surface area of the sample, and the Stefan constant. Generally, the higher the total normal emissivity of a material, the greater its radiated power. By measuring the total normal emissivity of a sample in the FIR range (refer to Appendix 1; Table S1), we can analyze the infrared properties of the sample. One can see that the total normal emissivity of the frame material before reinforcement, the frame material after reinforcement, and the reinforced point coating material is 0.86, 0.86, and 0.89, respectively, which is greater than that of the normal material and indicates that the frame material has higher radiation power. The radiation power of the reinforced frame material is similar to that of the pre‐strengthened frame material. The strengthening point coating material has the largest radiation power, indicating that the more FIR mineral content, the better the FIR performance of the material.

On the other hand, to further verify the FIR performance of the samples, we also obtained the infrared radiation spectra of the sample, as shown in (refer to Appendix 1; Figure S2). According to the results of the infrared radiation spectra, one can see that the peak value of the frame material corresponds to a wavelength of around 8 µm, which is in the FIR region. In addition, the peak value of reinforced point coating material is the largest, at about 8.5 W/ (cm^2^ µm). The peak value of the frame material before and after reinforcement is not much different, at about 8 W/ (cm^2^ µm), which is basically consistent with the test results of the total normal emissivity.

### 5.4 Outcomes

In accordance with the DED clinical examination procedure, all patients underwent a complete ophthalmic examination related to DED.

#### 5.4.1 Subjective symptoms

At the first, the completion of the OSDI and VAS questionnaires was performed prior to all examinations. Twelve questions comprised the ODSI questionnaire, which was subdivided into three subscales: vision‐related function, ocular symptoms, and environmental triggers. The score ranged from 0 (no symptoms) to 100 (severe symptoms).^59^ The VAS is a self‐reported scale. Subjects were asked to draw a vertical marker on a horizontal line to indicate their eye symptoms (including stickiness, burning/tingling, foreign body sensation, itching, blurred vision, photosensitivity, and pain), and the location of the marker indicated the degree of discomfort (0% for “no discomfort” and 100% for “maximum discomfort”).^61^

#### 5.4.2 Indicators of dry eye signs

TMH, which could value the detection of tear secretion, was acquired by the keratograph 5 M (K5M) (OCULUS, Wetzlar, Germany) and recorded in millimeters (mm). Conjunctival congestion was measured using the same machine, which can automatically produce a score. Using LipiView interferometer (TearScience, Morrisville, USA), LLT was analyzed based on a 20‐second video. After dripping a moistened aseptic fluorescein strip in the inferior fornix, a slit‐lamp microscope with a cobalt blue filter was used to record the time interval between the last blink and the appearance of the first random dry spot on the corneal surface, which is called FBUT. According to the American National Eye Institute/Industry scale (a total score of 0–15),^62^ CFS was scored by assessing the severity of ocular surface damage under slit lamp cobalt blue light after dyeing. When measuring SIT, patients were required to close their eyes for 5 min and the amount of wetting of the test strip was recorded.^63^

#### 5.4.3 Meibomian gland assessment

Using K5M (OCULUS, Wetzlar, Germany), the meibomian gland was evaluated according to the following criteria^20^: *Meibo‐Scan*: Level 0: no meibomian glands were absent; Level 1, 2, 3: missing area ≤1/3, 1/3–2/3, ≥2/3. *Eyelid Margin Signs*: One point was scored for each of the following eyelid margin signs: dull eyelid margin, eyelid margin thickening, eyelid margin hyperkeratinization, congestion at the front edge of the eyelid margin, hyperplasia of vessels around the meibomian mouth or telangiectasia. *Meibomian gland expressibility*: Five central glands of the upper and lower eyelid were evaluated separately. Grade 0: Secretions are extruded from all glands. Grade 1: Secretions are extruded from 3−4 glands; Grade 2: Secretions extruded from 1−2 glands; Grade 3: No secretion extruded from any gland. *Meibum quality*: 5 central glands of the upper and lower eyelid were evaluated separately. Grade 0: meibum is clear and transparent; Grade 1: meibum is cloudy; Grade 2: meibum is muddy with crumbs (particles); Grade 3: meibum is thick and toothpaste‐like.

#### 5.4.4 Safety evaluation

The School of Mathematical and Physical Sciences at Beijing University of Science and Technology conducted a safety study on this FIR eyeglass frame. The study included testing the chemical composition, spontaneous radiation characteristics, and radioactivity of the material.

During the implementation of this clinical trial, safety indicators were thoroughly assessed during each follow‐up visit, including measures such as VA, IOP, fundus photography, as well as detailed examinations of the periorbital skin (including edema, redness, and hair loss), eyelids, conjunctiva, sclera, cornea, iris, lens, vitreous, and other ocular structures using a slit lamp. These observations were conducted collaboratively by two experienced ophthalmologists from Beijing Tongren Hospital, ensuring a high level of authority and credibility.

### 5.5 Statistical analysis

Statistical analysis was performed with the SPSS version 26.0 (SPSS Inc., USA). Quantitative data are described statistically using the mean ± standard deviation (M ± SD), and rank data are described statistically using the median (interquartile spacing). For the outcome indicators, the Shapiro–Wilk test was used to estimate whether the measured variables obey distribution normality. The comparison of the patient's three follow‐up data was conducted using repeated measures analysis of variance. All statistical analyses were two‐sided, and statistical significance was set at *p* < 0.05.

## AUTHOR CONTRIBUTIONS

Lei Tian and Yihan Guo contributed equally to this work. *Methodology, writing—review & editing*: Lei Tian. *Formal analysis, writing—original draft*: Yihan Guo. *Data curation*: Silu Wang and Zhongying Li. *Conceptualization and supervision*: Ying Jie and Ningli Wang. All authors contributed to the article and approved the submitted version. All authors have read and approved the final manuscript.

## CONFLICT OF INTEREST STATEMENT

The authors declare that there is no conflict of interest regarding the publication of this paper.

## ETHICS STATEMENT

The study was conducted in accordance with the aims of the Declaration of Helsinki. The study involving human participants was reviewed and approved by the Ethics Committee of Beijing Tongren Hospital (board approval number: TREC2022‐KY005). The patients/participants provided their written informed consent to participate in this study.

## CLINICAL TRIAL REGISTRATION

This study is an interventional Clinical Trial, which has been registered in the Chinese Clinical Trial Registry (ChiCTR), and has obtained the clinical trial registration number: ChiCTR2300079099.

## Supporting information

Supporting Information

## Data Availability

The data that support the findings of this study are available from the corresponding author upon reasonable request.
