# Supplementary material for: Efficacy of far infrared functional glasses in the treatment of meibomian gland dysfunction‐related dry eye
Source: MedComm (2020). 2024 Mar 22;5(4):e507. doi: 10.1002/mco2.507 (PMC10959456; doi:10.1002/mco2.507)

**Efficacy of far infrared functional glasses in the treatment of meibomian gland dysfunction-related dry eye**

**Running Title:** The efficacy of far infrared functional glasses

Lei Tian^a,b,#^, Yihan Guo^a,#^, Silu Wang^a^, Zhongying Li^a^, Ningli Wang^a,*^, Ying Jie^a,*^

^a^. Beijing Institute of Ophthalmology, Beijing Tongren Eye Center, Beijing Tongren Hospital, Capital Medical University; Beijing Ophthalmology & Visual Sciences Key Laboratory, Beijing, China.

^b^. Beijing Advanced Innovation Center for Big Data-Based Precision Medicine, Beihang University and Capital Medical University, Beijing, China

*** Correspondence:**

Ningli Wang

Telephone: 010-13511026669

Fax: 58265922

Email: wningli@vip.163.com

Ying Jie,

Telephone: 010-13693572296

Fax: 58265900

Email: [jie_yingcn@aliyun.com](mailto:jie_yingcn@aliyun.com)

**Supplementary Information**

**Table 1. Test results of the normal full emissivity of the frame material before reinforcement, the frame material after reinforcement, and the reinforcing point coating material**

| **Sample** | **Test item** | **Test result** | **Test instrument** |
| --- | --- | --- | --- |
| **Frame material before reinforcement** | Total normal emissivity | 0.86 | GB/T 7287-2008 |
| **Frame material after reinforcement** | Total normal emissivity | 0.86 | GB/T 7287-2008 |
| **Reinforced point coating material** | Total normal emissivity | 0.89 | GB/T 7287-2008 |

**Figure S1. YDkai "Light of life" FIR Functional glasses**


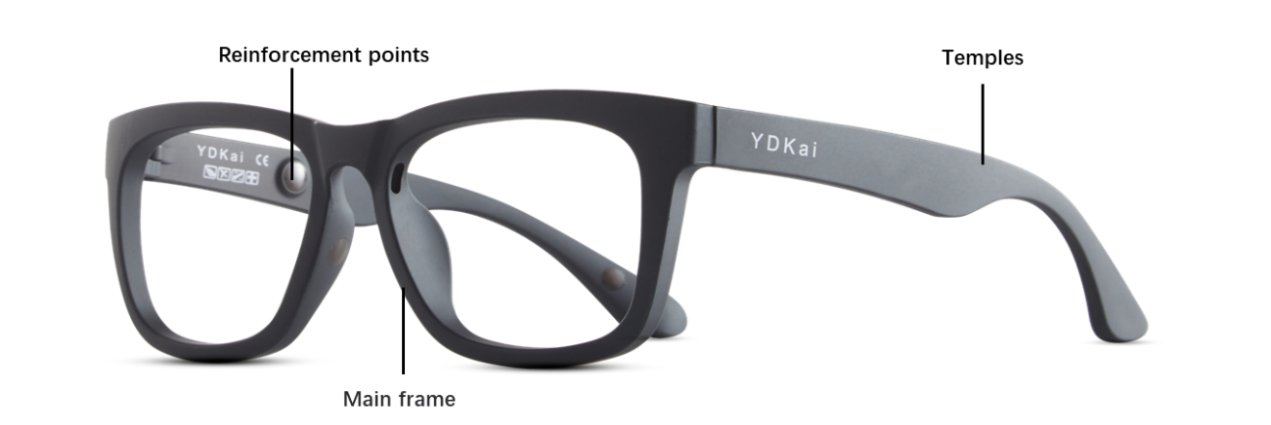


**Figure S2. Infrared radiation spectrum of the frame material at an average temperature of 100℃. The radiant emittance** $\boldsymbol{M}_{\boldsymbol{\lambda}}\mathbf{(}\boldsymbol{T}\mathbf{)}$ **(W/(cm^2^·μm)) as a function of wavelength (μm) for the frame material before reinforcement (A), the frame material after reinforcement(B), and reinforced point coating materials (C), respectively.**


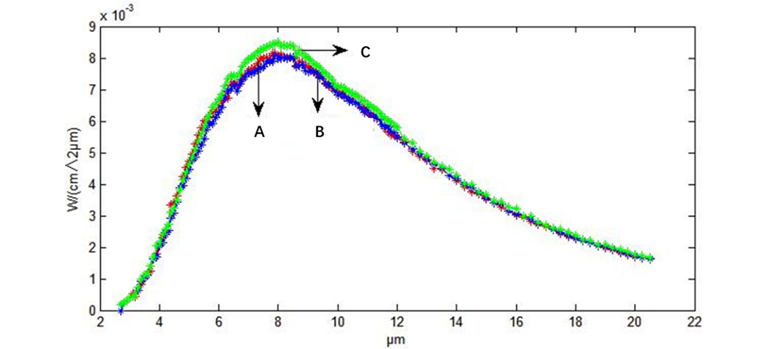

Supplement: Supplementary file 1 — Supporting Information [file MCO2-5-e507-s001.docx]
